# Essential oil composition analysis of three cultivars seeds of *Resina ferulae* from Xinjiang, China

**DOI:** 10.4103/0973-1296.80668

**Published:** 2011

**Authors:** Xiaojin Li, Yue’e Wang, Jun Zhu, Qiong Xiao

**Affiliations:** 1 *Chinese Medicine and National Medicine Research Institute of Xinjiang Uighur Autonomous Region, Urumqi 830002*; 2 *College of TCM, Xinjiang Medical University, Urumqi 830011, China*

**Keywords:** Essential oil, gas chromatography-mass spectrometry, *Resina ferulae*

## Abstract

**Objective:**

Three cultivars seeds of *Resina ferulae* were analyzed for essential oil composition, *Ferula sinkiangensis K. M. Shen, Ferula fukangensis K. M. Shen,* and *Ferula ovina*, investigated differences among different genera of medicinal *R. ferulae*.

**Materials and Methods:**

The essential oils were extracted by the method of hydrodistillation and analyzed by gas chromatography-mass spectrometry (GC-MS), using normalization method to calculate relative amount.

**Results:**

Twenty-six compounds were identified in *F. sinkiangensis K. M. Shen*, comprised 99.001% of total essential oil; 21 compounds were identified in *F. fukangensis K. M. Shen,* comprised 100% of total essential oil; 25 compounds were identified in *F. ovina*, comprised 99.459% of total essential oil. n-Propyl sec-butyl disulfide is the main component in three cultivars seeds of *R. ferulae,* accounting for 55.875%, 49.797%, 53.781%, respectively.

**Conclusion:**

Little diversity among three cultivars seeds of *R. ferulae* from Xinjiang.

## INTRODUCTION

*Ferula sinkiangensis K. M. Shen* is a genus of the Umbelliferae that used in TCM for a long time by China, and grow in Xinjiang of China. The resin from *F. sinkiangensis K. M. Shen* and *Ferula fukangensis K. M. Shen* are recorded in “Pharmacopoeia of People’s Republic of China”[1] for combating malaria, treating dysentery, counteracting toxins, deodorant, killing parasitic worms, removing stagnation, resolving phlegm, and others. The resin and roots are used as conventional drugs for Xinjiang people, especially essential oil as its major active components.[2] In the recent years, foreign researches on *Resina ferulae* are mainly focusing on the findings of plant estrogen active composition and anticancer substances.[3] The local people in Xinjiang use the roots to replace the resin, but there is no research on mechanisms of its effects and chemical composition of R. *ferulae* seeds. This article aims to study diversity among three cultivars seeds of medicinal *R. ferulae* by analyzing essential oil composition. In addition, provide foundation of basic research to found perfect species classification.

## MATERIALS AND METHODS

### Plant materials and reagents

The three cultivars seeds of *R. ferulae*, including *F. sinkiangensis K. M. Shen, F. fukangensis K. M. Shen,* and *F. ovina*, were collected from Yili, Fukang, and Altay of Xinjiang in July 2008, respectively. It was identified by Xiaojin Li, a researcher and Guoping Wang, a research assistant of Chinese Medicine and National Medicine Research Institute of Xinjiang Uighur Autonomous Region. Voucher specimens were deposited in the herbarium room of Xinjiang Chinese Medicine and National Medicine Research Institute.

HP6890GC/5973MSD GC-MS Spectrometry (America Hewlett-Packard Co. Ltd.); anhydrous sodium sulfate, and others are all from domestic analytical reagent (AR).

### Essential oil composition analysis

Three cultivars seeds (100 g each) extracted by hydrodistillation[4] and dried over anhydrous sodium sulfate for 12 h, then stored the light yellow transparent oil under refrigeration prior to analysis.

Gas chromatography condition: HP6890GC/5973MSD GC-MS Spectrometry, HP-5MS capillary column (30 m × 0.25 mm × 0.25 µm), the temperature program was 60°C–280°C at a rate of 10°C/min, and injector temperature was 280°C. The injected volume was 1 µL, helium was used as carrier gas, flow rate 1 mL/min; gas chromatography-mass spectrometry analyses were performed in the EI mode 70 eV, scan range 15–550 m/z, scan time 0.5 s. Ion source temperature was 230°C, ionization mode EI, voltage multiplier 0.7 kV, deduct chromatogram of solvent peak then the rest of the total peak areas as 100%, using normalization method to calculate relative amounts of individual components.

The chemical structure was searched using MS for each chromatography peak by NIST 05. L.

## RESULTS AND DISCUSSION

The seeds of three cultivars of *R. ferulae* were reduced to coarse powders and the essential oils were extracted by hydrodistillation; and the yields of essential oil were 3.8% for *F. sinkiangensis K. M. Shen,* 1.2% for *F fukangensis K. M. Shen,* and 1.8% for F *ovina*. The essential oil contents for three cultivars seeds of *R. ferulae* were high, have better use value.

Meanwhile, 26 compounds were identified in *F sinkiangensis K. M. Shen,* comprised 99.001% of the total essential oil; 21 compounds were identified in *F fukangensis K. M. Shen*,comprised 100% of total essential oil; 25 compounds were identified in *F ovina,* comprised 99.459% of total essential oil. The results are presented in Table 1.

**Table 1:** Chemical composition of essential oil of seeds from Ferula sinkiangensis K. M. Shen, Ferula fukangensis K. M. Shen, and Ferula ovina

Ferula fukangensis K.	M. Shen and Ferula ovina	Ferula sinkiangensis K. M. Shen	Ferula fukangensis K. M. Shen	Ferula ovina			
No	Compounds	Retenti On time (min)	Area %	Retenti On time (min)	Area %	Retenti On time (min)	Area
1	Disulfide,bis(1-methylpropyl)	8.2	3.383	8.17	2.115	8.182	3.217
2	n-Propyl sec-butyl disulfide	7.464	55.875	7.482	49.797	7.458	53.781
3	β-Pinene	4.696	0.331	4.856	0.425	4.856	0.042
4	3,7-dimethyl-1,3,6-Octatriene	5.538	0.722	5.538	0.636	5.698	0.247
5	1,3,6-Octatriene, 3,7-dimethyl-,(Z)-	5.745	1.363	5.769	2.072		
6	Disulfide,bis[1-(methylthio)ethyl]	10.014	0.214			10.678	2.239
7	IH-Cycloprop[elazulene,decahvdro-1,1,7-trimethyl-4-methylene-,[laR-(la.a.,4a.	12.789	4.299			11.804	0.614
8	13.,7.alpha.,7a.beta.,7b.alpha.)]-1 H-Cyclopropa[a]naphthalene, 1a,2,3,3a.4.5,6, 7b-octahydro-1,1,3a, 7-tetramethyl-,[1aR-(la.a.,3a.a.,7b.a.)]	13.565	3.131	13.571	2		
9	1-(p-Tolyl)-2-imidazoIidinone			8.929	15.547	8.757	1.14
10	Benzene.(2-methylbutyl)-	5.852	2.092				
11	Disulfide,ethyl 1-methylpropyl	6.125	0.203				
12	5,6-Dihydro-2-methyl-l,4-dithiine-3-carboxylic acid	8.532	2.133				
13	5,6-Dihydro-2-methyl-I,4-dithiine-3-carboxylic acid	8.751	1.116				
14	Cyclohexadiene,1-methyl-4-(1-methyl ethyl)	4.589	0.047				
15	Disulfide, bis[1-(methylthio)ethyl]	10.103	1.046				
16	1,2,4-Methenoazulene,decahydro-I,5,5,8a-tetramethyl-,[I S-(l.alpha.,2.alpha.,3a.beta.,4.a.,8a.0.,9R*)]	10.435	0.204				
17	Methylcis-2-trimethylsilylcyclopropane-I-carboxylate	11.182	17.185				
18	1,6,10-Dodecatriene,7,11-dimethyl-3-methylene-,(Z)	11.496	0.412				
19	Cyclohexene,l-methyl-4-(5-methyl-Imethylene-4-hexenyl)-,(S)	12.089	1.865				
20	cis-,a.-Bisabolene	12.492	0.442				
21	1 R-.a.-Pinene (I R-a)	4.103	1.19				
22	Guaiol	13.257	0.526				
23	Disulfide,dipropyl	6.64	0.149				
24	Hinesol	13.719	0.313				
25	Neoisolongifolene	13.838	0.855				
26	Bicyclo[4.4.0]dec-l-ene,2-isopropyl-5-methyl-9-methylene	13.903	0.689				
27	Disulfide,dipropyl	6.978	0.216				
28	2H-Pyran,tetrahydro-4-methyl-2-(2-methyl-1-propenyl)			6.652	0.156		
29	2,4,6-Octatriene,2,6-dimethyl-,(E.Z)-			6.961	0.303		
30	2,4,6-Octatriene,2,6-dimethyl-,(E,Z)-			7.133	0.126		
31	Estragole			7.998	0.334		
32	6-Octen-l-ol,3,7-dimethyl-,(R)-			8.413	0.578		
33	Benzene,1-methoxy-4-methyl-2-(1-methylethyl)	8.603	0.189				
34	Dimethyl trisulfide	4.672	0.062				
35	Aceticacid,1,7,7-trimethylbicyclo[2.2.1]hept-2-yl ester	9.202	0.291				
36	2,6-Octadiene,2,6-dimethyl-	10.04	2.584				
37	Methyl 2-(methylthio)butyrate	11.223	17.603				
38	gamma.-Elemenea	12.789	2.243				
39	Naphthalene,l,2,3,5,6,7,8,8aoctahydro-1,8a-dimethyl-7-(Imethylethenyl)-,[]S-(l.a.,7.a.,8a.a.)]	13.731	1.016				
40	Neoisolongifolene	13.838	0.922				
41	Guaiol	13.927	1.001				
42	1,3-Cyclohexadiene,1-methyl-4-(1-methylethyl)			4.571	0.231		
43	Methyl sec-butyl disulphide			4.968	0.059		
44	3-(Methylthio)-2-butanone			10.127	1.132		
45	1,2,4-Methenoazulene,decahydro-1,5,5,8a-tetramethyl-,[1S-(1.a,2.a.,3a.beta.,4.a.,8a.0,9R*)]			10.435	0.904		
46	Naphthalene,1,2,3,4.4a,5,6,8aoctahydro-7-methyl-4-methylene-1-(1-methylethyl)-,(I.a.,4a.a.,8a.a.)			10.814	0.121		
47	1,4-Methanoazulene,decahydro-4,8,8-trimethyl-9-methylene-,[IS-(l.a.,3a.β.,4.a.,8a.β.)]-			10.897	0.409		
48	Caryophyllene			11.051	1.805		
49	Thiazole,tetrahydro-			11.182	24.009		
50	1H-Benzocycloheptene,2,4a,5,6,7,8,9,9a-octahydro-3,5,5-trimethyl-9-methylene-.(4aS-cis)			11.466	0.393		
51	.a.-Caryophyllene			11.508	0.389		
52	Thiopropionamide			11.662	1.849		
53	Longifolene-(V4)			11.87	0.716		
54	Naphthalene,1,2,3,4,4a,5,6,8aoctahydro-4a,8-dimethyl-2-(l-methyl ethenyl)-,[2R-(2.a.,4a.a.,8a.13.)]			11.917	0.532		
55	Seychellene			12.036	0.345		
56	1 H-Benzocycloheptene,2,4a,5,6,7,8-hexahy dro-3,5,5,9-tetramethyl-,(R)			12.083	1.106		
57	1,6,10-Dodecatrien-3-ol,3,7,11-trimethyl-.[S-(Z)]			12.747	1.08		
58	Famesene epoxide,E-			13.381	0.95		
59	Di-epi-.a.-cedrene-(I)			13.909	2.689		

The result showed that there has been an apparent diversity among the three cultivars seeds of medicinal *R. ferulae*, according to the analysis from yields of essential oil and essential oil composition.

At the same time, three cultivars seeds of *R. ferulae* have strong smell, like the odor of garlic. The experiment result showed that the three cultivars seeds have high content of sulfur compounds, which is similar to some earlier findings. The sulfur compounds from the seeds are disulfide and trisulfide, finding tetrasulfide disulfide, bis[1-(methylthio) ethyl] in *F ovina,* which pharmacological effects need further research.

The category of chemical compounds in the essential oil of the three cultivars seeds are showed in Table 2, n-Propyl sec-butyl disulfide is the main component (55.88 %,49.80 %, 53.78 %) for each oil. Propyl sulfide can act as an antimutagenic and anticancer agent, and protect the immune system and cardiovascular system. Moreover, it can act as an antitumor, antifungal, and antiparasitic agent, and also it can activate liver detoxification enzyme system and inhibit the toxins produced by bacteria and viruses.[5] Terpenoids, mostly antioxidants, are anti-cancer agents, prevent the formation of cholesterol, promote the protection of the enzyme, and so on.[6] So the effects of essential oil of the three cultivars seeds from *R. ferulae* needs further research.

**Table 2:** The category of chemical compounds in the essential oils of the three cultivars seeds from *Resina ferulae*

Name	Monoterpenoids	Sesquiterpenoids	Polysulfide alkanes	Others *Ferula sinkiangensis K.*
M. Shen	3.65%	8.34%	64.34%	23.68%
*Ferula ukangensis K.*	6.636%0	5.321%0	68.515%0	18.528%0
M. Shen				
*Ferula ovina*	0.52%	8.26%	86.29%	4.94%

Table 2 shows the perfect species classification according to the category of chemical compounds in the essential oils of the three cultivars seeds from *R. ferulae*. Also is reliable evidence of clinical utilization of the three cultivars seeds from *R. ferulae*. At the same time, provide further research foundation and methods of exploring which composition play an important role in its effects among the three cultivars seeds from *R. ferulae*, according to the analysis of composition.
